# Studies of a Ring-Cleaving Dioxygenase Illuminate the Role of Cholesterol Metabolism in the Pathogenesis of *Mycobacterium tuberculosis*

**DOI:** 10.1371/journal.ppat.1000344

**Published:** 2009-03-20

**Authors:** Katherine C. Yam, Igor D'Angelo, Rainer Kalscheuer, Haizhong Zhu, Jian-Xin Wang, Victor Snieckus, Lan H. Ly, Paul J. Converse, William R. Jacobs, Natalie Strynadka, Lindsay D. Eltis

**Affiliations:** 1 Department of Biochemistry and Molecular Biology, University of British Columbia, Vancouver, British Columbia, Canada; 2 Howard Hughes Medical Institute, Albert Einstein College of Medicine, Bronx, New York, United States of America; 3 Department of Chemistry, Queen's University, Kingston, Ontario, Canada; 4 Department of Microbial and Molecular Pathogenesis, Texas A&M University Health Science Center, College Station, Texas, United States of America; 5 Center for Tuberculosis Research, Department of Medicine, Johns Hopkins University, Baltimore, Maryland, United States of America; 6 Department of Microbiology and Immunology, University of British Columbia, Vancouver, British Columbia, Canada; University of Washington, United States of America

## Abstract

*Mycobacterium tuberculosis*, the etiological agent of TB, possesses a cholesterol catabolic pathway implicated in pathogenesis. This pathway includes an iron-dependent extradiol dioxygenase, HsaC, that cleaves catechols. Immuno-compromised mice infected with a Δ*hsaC* mutant of *M. tuberculosis* H37Rv survived 50% longer than mice infected with the wild-type strain. In guinea pigs, the mutant disseminated more slowly to the spleen, persisted less successfully in the lung, and caused little pathology. These data establish that, while cholesterol metabolism by *M. tuberculosis* appears to be most important during the chronic stage of infection, it begins much earlier and may contribute to the pathogen's dissemination within the host. Purified HsaC efficiently cleaved the catecholic cholesterol metabolite, DHSA (3,4-dihydroxy-9,10-seconandrost-1,3,5(10)-triene-9,17-dione; *k*_cat_/*K*_m_ = 14.4±0.5 µM^−1^ s^−1^), and was inactivated by a halogenated substrate analogue (partition coefficient<50). Remarkably, cholesterol caused loss of viability in the Δ*hsaC* mutant, consistent with catechol toxicity. Structures of HsaC:DHSA binary complexes at 2.1 Å revealed two catechol-binding modes: bidentate binding to the active site iron, as has been reported in similar enzymes, and, unexpectedly, monodentate binding. The position of the bicyclo-alkanone moiety of DHSA was very similar in the two binding modes, suggesting that this interaction is a determinant in the initial substrate-binding event. These data provide insights into the binding of catechols by extradiol dioxygenases and facilitate inhibitor design.

## Author Summary

*Mycobacterium tuberculosis*, the etiological agent of TB, is the most devastating infectious agent of mortality worldwide: it is carried by one-third of all humans and kills nearly two million people annually. Recent work has established that the pathogen metabolizes cholesterol, although the role of this metabolism in pathogenesis remains unclear. In the current study, we demonstrate that HsaC is a key enzyme in the cholesterol catabolic pathway and that it can be inactivated by compounds that resemble its substrate. Using molecular genetic approaches, we demonstrated that the enzyme is essential for the growth of *M. tuberculosis* on cholesterol and that a lack of this enzyme impairs the survival of the pathogen in each of two animal models. These studies provide definitive evidence that *M. tuberculosis* metabolizes cholesterol during infection and that this metabolism occurs during the early stages of infection. The oxygen-utilizing enzymes of the cholesterol catabolic pathway, of which HsaC is but one example, are intriguing potential chemotherapeutic targets, as their inhibition can lead to toxic metabolites, including reactive oxygen species. Overall, our study combines a variety of approaches to provide novel insights into a disease of global importance and into the mechanism of an interesting class of enzymes.

## Introduction

*Mycobacterium tuberculosis*, the leading cause of mortality among bacterial pathogens, infects one-third of the human population and is responsible for approximately 2 million deaths annually. The global threat of TB has risen alarmingly due to two factors: the bacterium's deadly synergy with HIV [1] and the emergence of multidrug-resistant strains, including extensively drug-resistant strains (XDR-TB) that are virtually untreatable with current chemotherapies [2]. An important factor that contributes to the disease's prevalence is the pathogen's unusual ability to survive for long periods of time, and even to replicate, in the macrophage [1]. The mechanisms by which *M. tuberculosis* persists in the macrophage remain largely unknown, but such mechanisms are good targets for novel therapeutic agents.

**Figure 1:**
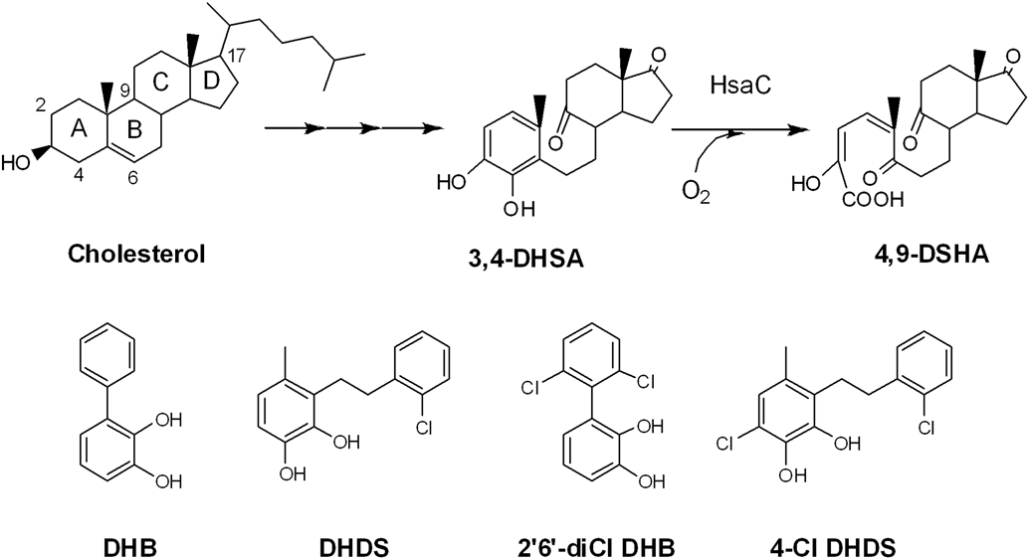
The role of HsaC in the cholesterol degradation pathway. Cholesterol is transformed to DHSA (3,4-dihydroxy-9,10-seconandrost-1,3,5(10)-triene-9,17-dione) via multiple enzymatic steps. HsaC catalyzes the extradiol ring-cleavage of DHSA to DSHA (4,5-9,10-diseco-3-hydroxy-5,9,17-trioxoandrosta-1(10),2-diene-4-oic acid).

A suite of genes critical for survival of *M. tuberculosis* in the macrophage [3] was recently discovered to be involved in cholesterol degradation [4]. As in the aerobic bacterial degradation of other steroids, the core 4-ringed structure is degraded via opening of ring B with concomitant aromatization of ring A. The resulting phenolic metabolite is hydroxylated, yielding a catechol, 3,4-dihydroxy-9,10-seco-nandrost-1,3,5(10)-triene-9,17-dione (DHSA). HsaC catalyzes the *meta*-cleavage of DHSA to produce 4,9-DSHA (4,5-9,10-diseco-3-hydroxy-5,9,17-trioxoandrosta-1(10),2-diene-4-oic acid; Figure 1). Recent work by Pandey and Sassetti [5] indicates that *in vitro*, the pathogen uses different parts of the cholesterol molecule for energy and the biosynthesis of phthiocerol dimycocerosate (PDIM), a virulence-associated lipid, respectively. Using a mutant defective in the *mce4*-encoded cholesterol transporter [6], Pandey and Sassetti further demonstrated that cholesterol uptake is essential for persistence in the lungs of chronically infected mice and for growth in IFN-γ-activated macrophages that predominate during the chronic phase of the illness. However, this deletion impaired *in vitro* growth on cholesterol only modestly.

HsaC shares ∼40% amino acid sequence identity with BphC (EC 1.13.11.39), a well-characterized type I extradiol dioxygenase that cleaves 2,3-dihydroxybiphenyl (DHB) and that is potently inhibited by 2′,6′-diCl DHB [7], a polychlorinated biphenyl metabolite (Figure 1). Extradiol dioxygenases typically utilize Fe(II) in a 2-His 1-carboxylate facial triad coordination environment to catalyze the cleavage of catechols and their analogues. In the proposed mechanism, based on biochemical, spectroscopic, kinetic and structural studies [8],[9],[10], the catecholic substrate binds first to the enzyme's Fe(II) center in a bidentate manner, displacing two solvent ligands. Thus activated, the ferrous center binds O_2_, leading to the formation of an Fe(II)–bound alkylperoxo intermediate. The latter undergoes heterolytic O-O bond cleavage and Criegee rearrangement involving 1,2-alkenyl migration to produce a lactone intermediate. Hydrolysis of the latter affords the ring-cleaved product. Several of the proposed intermediates were recently substantiated in structural studies of homoprotocatechuate 2,3-dioxygenase (HPCD) and a slow substrate, 4-nitrocatechol [11]. Nevertheless, some steps of the catalytic cycle remain unclear, including the multi-step binding of the catecholic substrate [12].

We report herein studies of HsaC from *M. tuberculosis* H37Rv. An *hsaC*-null gene deletion mutant was generated and tested in liquid culture and in animal models to assess the role of HsaC in cholesterol degradation and pathogenicity. The specificity of the enzyme was investigated, and crystal structures of HsaC were obtained in its substrate-free form and in complex with the steroid metabolite, DHSA. The results provide insights into the binding of catechols to extradiol dioxygenases and the role of cholesterol metabolism in pathogenesis.

## Results

### Substrate preference and inactivation of HsaC

To characterize HsaC from *M. tuberculosis* H37Rv, we anaerobically purified the enzyme to >99% apparent homogeneity from a recombinant *E. coli* strain. Purified enzyme contained 0.92 equivalents of iron. To stabilize HsaC for steady state kinetic assays, the enzyme was diluted in 20 mM HEPES, 80 mM NaCl, pH 7.5 supplemented with 5% *t*-butanol, 2 mM dithiothreitol, 0.1 mg/ml bovine serum albumin and stored on ice under an inert atmosphere. Due to the oxidative inactivation of both the enzyme and DHSA in air-saturated buffer, kinetic studies were performed using buffer equilibrated with 5% oxygen in nitrogen to obtain better quality data.

Steady-state kinetic studies revealed that HsaC has 90-times greater specificity for the steroid metabolite, DHSA, over DHB, the preferred substrate of BphC (Table 1). To facilitate further kinetic characterization of HsaC, we designed a substrate analogue, DHDS (Figure 1), which incorporated potentially important features of DHSA including the methyl group on the catecholic ring and a saturated 2-carbon bridge between the two ring systems. The specificity of HsaC for DHSA was 10-times greater than for DHDS. 2′,6′-diCl DHB, a PCB metabolite that potently inhibits (7±1 nM) and oxidatively inactivates BphC [7], and 4-Cl DHDS, a chlorinated substrate analogue were cleaved very slowly by HsaC (partition ratios<50). While the *K*_m_ values of HsaC for the PCB metabolite and the chlorinated steroid metabolite were ∼1,000-fold greater than those of BphC, both compounds have clear potential as competitive inhibitors of the mycobacterial dioxygenase. The steady-state utilization of O_2_ by HsaC was evaluated in the presence of DHDS due to the ease of preparation of this compound. We anticipate that the reactivity of the enzyme with O_2_ will be very similar in the presence of DHDS and DHSA as the two compounds have similarly substituted catecholic rings. The apparent *K*_mO2_ of HsaC was 90±20 µM: 13-fold less than that of BphC [13] and nearly 3-times less than the concentration of O_2_ in air-saturated buffer. Nevertheless, the specificity of HsaC for O_2_ is only 5-times less than that of BphC (0.20±0.01 µM^−1^ s^−1^ *vs.* 1.0±0.1 µM^−1^ s^−1^).

**Table 1:** Steady-state kinetic and inactivation parameters of HsaC for various catecholic substrates.

Compound	*K*_m_ (µM)	*k*_cat_ (s^−1^)	*k*_cat_/*K*_m_ (µM^−1^ s^−1^)	Partition Ratio
DHB	10 (2)	1.6 (0.1)	0.16 (0.02)	1,060 (10)
DHDS	4.8 (0.6)	6.7 (0.2)	1.4 (0.1)	2,300 (300)
DHSA	1.1 (0.2)	15.9 (0.6)	15 (2)	1,900 (200)
2′,6′-diCl DHB	6.7 (0.7)	6.6 (0.3)×10^−3^	0.99 (0.04)×10^−3^	30 (10)
4-Cl DHDS	3.2 (0.3)	277 (9)×10^−3^	85 (6)×10^−3^	44 (4)

Experiments were performed using 20 mM HEPES, 80 mM NaCl, pH 7.0 (I = 0.1) equilibrated with 5% O_2_ at 25°C. Values in parentheses are standard errors.

Extradiol dioxygenases are subject to oxidative inactivation during catalytic turnover [14]. Accordingly, we investigated the susceptibility of HsaC to inactivation during the steady-state cleavage of each of the catecholic substrates using the partition ratio, the amount of substrate consumed per mole of enzyme inactivated. As reported for BphC, HsaC was more susceptible to inactivation by poorer substrates (Table 1). Nevertheless, the observed partition ratios are more than 2 orders of magnitude less than what has been reported for other extradiol dioxygenases for their preferred substrates [14],[15]. Finally, 2′,6′-diCl DHB inactivated HsaC with a partition ratio similar to that in BphC (<50).

### Structures of HsaC in complex with DHSA

**Figure 2:**
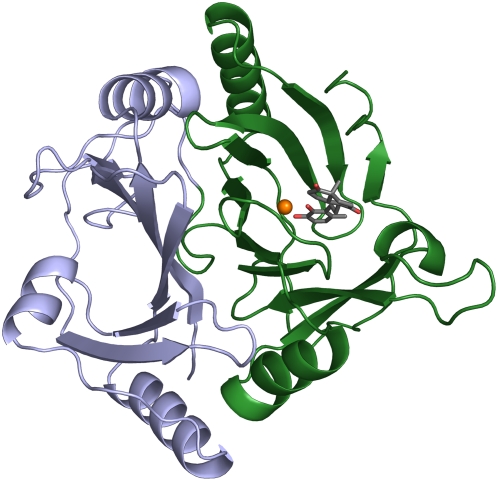
The structural fold of HsaC, molecule A (DHSA bidentate bound). The Cα traces of the structurally similar N- and C-terminal domains are colored in silver and dark green, respectively. As in other two-domain type I extradiol dioxygenases, the active site is located in the C-terminal domain. The iron ion is colored orange. The C and O atoms of the bound DHSA are grey and red, respectively.

Crystal structures of HsaC were obtained in its substrate-free form and in complex with DHSA at resolutions of 2.0 and 2.2 Å, respectively. The asymmetric unit of the crystals contains two well-ordered molecules. Crystallographic four-fold symmetry of the two molecules in the asymmetric unit indicates the enzyme is octameric, like BphC. Crystallographic statistics are summarized in Table 2. The overall fold of HsaC is that of a two-domain type I extradiol dioxygenase, with the structure most closely resembling that of BphC [16] (rmsd of 1.16 Å for the 275 common Cα atoms; Figure 2). The active site is located within the central cavity of the slightly larger C-terminal domain, with the catalytically essential mononuclear Fe^2+^ ligated by His145, His215 and Glu266. In the resting state enzyme, the coordination sphere is completed by two solvent molecules (wat1 and wat2) such that the metal ion's coordination geometry is square pyramidal. The metal-ligand distances (Table S1) and ligand-metal-ligand angles (Table S2) are within experimental error of those observed in BphC [16].

**Table 2:** Crystallographic properties, X-ray diffraction data, and refinement statistics for HsaC.

Diffraction Data	HsaC (substrate free)	HsaC:DHSA
X-ray source	Cu-Kα	ALS 8.2.2
Wavelength (Å)	1.542	1.000
Space group	*P*42_1_2	*P*42_1_2
Unit cell (Å)	*a* = *b* = 123.7, *c* = 106.7	*a* = *b* = 124.3, *c* = 106.3
Resolution (Å)	2.0	2.2
Highest shell (Å)	2.0–2.2	2.32–2.20
Total observations	791,882 (109,166)	384,012 (55,996)
Unique reflections	56,454 (8,105)	42,904 (6,156)
*I/σI*	28.9 (7.1)	26.0 (6.1)
R_sym_(%)‡	9.1 (44.1)	8.1 (38.9)
Completeness (%)	99.9 (99.9)	100 (100)
Refined Model		
Resolution range (Å)	20–2.0	20–2.2
No. Reflections	53,536	40,622
R_free_/R_factor_ (%)†	22/18	26/19
No. atoms:		
Total	5,364	5,224
Protein	4,677	4,692
Solvent	712	485
Mean B values (Å^2^):		
Protein	19.4	25.1
Fe	14.8	20.3
DHSA	-	45.5
R.m.s. deviations:		
Bond lengths (Å)	0.015	0.024
Bond angles (deg)	1.67	2.42

**‡:** *R*_sym_ = Σ_h_Σ_i_ I(hkl)−〈I(hkl)〉/Σ_h_Σ_i_I(hkl).

**†:** *R*_work_ = Σ||*F*_obs_|−|*F*_calc_||/Σ|*F*_obs_|. *R*_free_ is the *R*_work_ value for 5% of the reflections excluded from the refinement. Data for the highest resolution shell are given in parentheses.

The two most significant structural differences between HsaC and BphC appear to be associated with the larger substrate-binding pocket of the former (550 Å^3^ in HsaC versus 420 Å^3^ in BphC, as calculated by VOIDOO [17]). First, the loop-helix-loop segment comprising residues 172–190 in HsaC, which contributes to the external wall of the substrate-binding pocket, angles outwards and contains a 6-residue insertion with respect to BphC, increasing the opening of the substrate-binding pocket by up to 10 Å. Second, the distal portion of the substrate-binding pocket, which accommodates the non-catecholic portion of the substrate, is lined with fewer bulky residues in HsaC. For example Met175, Phe202, His209 in BphC (PDB 1HAN) are Leu174, Met207 and Val214 in HsaC. Both the insertion and the smaller residues occur in other steroid-degrading extradiol dioxygenases [4].

**Figure 3:**
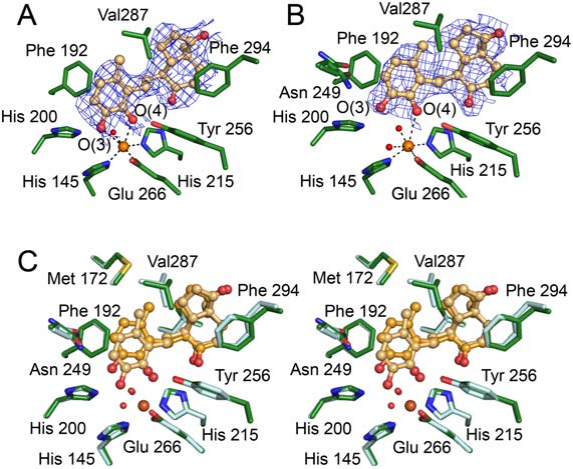
The binding modes of DHSA in HsaC. (A) DHSA in molecule A. (B) DHSA in molecule B. The (2*F*_o_−*F*_c_) electron density (blue, contour level = 1σ) was calculated without ligand to remove bias. (C) Stereo view of a structural superposition of DHSA in the two molecules in the asymmetric unit. HsaC:DHSA in molecule A is colored in dark green/yellow. HsaC:DHSA in molecule B is colored in light green/orange.

In crystals of HsaC soaked anaerobically with DHSA, the active site cavity of each molecule in the asymmetric unit contained additional electron density that corresponds to the steroid metabolite, DHSA (Figure 3). The structure of the protein in the two molecules is essentially identical to that of the substrate-free enzyme (rmsd of 0.39 Å for the 597 common Cα atoms) except as noted below. However, in both molecules, the iron is hexacoordinate with a distorted octahedral geometry instead of being pentacoordinate with square pyramidal geometry as in the resting state enzyme. Remarkably, DHSA binds in different modes in each of the molecules, with the catecholic ring coordinating the Fe in bidentate and monodentate manners in molecules A and B, respectively (Table 3). The sites appear to be relatively well ordered in each molecule. This interpretation is supported by ligand-omit and *F*_o_−*F*_c_ difference maps calculated using phases derived from the model in the absence of any ligands. These maps indicate that the electron density was compatible with the active sites of molecules A and B being fully occupied with DHSA (Figure S1), although the latter is slightly less ordered in molecule B. To further rule out alternate interpretations of the electron density in molecule B, the density was also fit using DHSA in a second orientation (rotated 180 degrees upon the substrate's orthogonal axis) and the product, DSHA. After refinement, these trials yielded strong positive residual density in proximity to the iron and very high temperature factors around the catechol ring, indicating that the current refined models are correct.

**Table 3:** Histopathology scores of lung tissue of guinea pigs infected with Δ*hsaC* mutant.

Strain	Week	# granulomas	#fields	#granulomas/field	Score^a^
H37Rv	4	18±8^b^	2.25±0.4	8±2.5	1.7±0.4
	8	27±3	2.25±1.8	12±11.8	1.8±0.4
Δ*hsaC*	4	1.5±2***	1±0	1.5±2.1	0.4±0.2
	8	2.5±1***	1.5±0.4	1.8±0.9**	0.3±0.1
Δ*hsaC* Comp	4	10.5±1†	2±0	5.3±0.4	1.8±0
	8	28.5±2†††	1.75±0.4	16.5±2.1††	2.5±0.7

a Scale of 0–4 ([39]).

b Mean±sd of 2 guinea pigs per time point (*p<0.05, **p<0.01, ***p<0.001 Δ*hsaC* compared to H37Rv).

**†:** p<0.05.

**††:** p<0.01.

**†††:** p<0.001 Δ*hsaC*-Comp compared to Δ*hsaC*.

The asymmetric, bidentate binding of the catecholic moiety in molecule A (Figure 3) corresponds to that which has been reported in other extradiol dioxygenase:substrate complexes [11],[15]. Briefly, the proximal hydroxyl (O4) of the catecholic ring binds the Fe in the site *trans* to His145 and the distal hydroxyl (O3) binds *trans* to His215, displacing the two water molecules in the resting state enzyme (Figure 3A). The coordination sphere is completed by a solvent species (wat267) *trans* to Glu266, presumed to occupy the O_2_-binding site. The catecholic moiety is bound asymmetrically to the iron in the sense that the O3-Fe distance is longer than that of O4-Fe, consistent with the monoanionic nature of catechol, as observed in BphC and homoprotocatechuate 2,3-dioxygenase (HPCD). Other hydrogen bonds involving the catecholic hydroxyls reported in BphC are conserved in HsaC.

In molecule B, the catecholic ring is bound to the iron in a monodentate manner via the 4-hydroxyl (proximal) group with an O4-Fe distance of 2.8 Å (Figure 3B). The 3-hydroxyl group forms a long hydrogen bond with Asn249 (3.0 Å) and a water molecule that is coordinated to the metal instead of with Asp250 as in the bidentate binding mode. With respect to its conformation in molecule A, the catecholic moiety of DHSA is rotated 60° clock-wise around the ligand's C6–C7 bond such that the O3 hydroxyl is 3.7 Å away from the Fe. The DHSA has greater temperature factors (mean 51 Å^2^) in molecule B than in molecule A (mean 35 Å^2^), consistent with a greater degree of disorder and lesser binding affinity of the monodentate-bound catechol versus the bidentate-bound molecule.

In contrast to the different binding modes of the catecholic ring in the two molecules, the bicycloalkanone moiety of the bound DHSA occupies strikingly similar conformations in the two complexes, suggesting that this moiety is a major determinant in the binding of the substrate. More precisely, the bicycloalkanone moiety occupies a largely hydrophobic portion of the substrate-binding site, contacting Leu174, Leu190, Leu205, Val 214, and Phe294 (Figure 3C). These five residues are conserved in extradiol dioxygenases known or thought to preferentially cleave DHSA [4]. In both molecules, the carbonyl oxygen at C9 is orientated towards the iron ligand His215 (O9) while that at C17 interacts with up to three ordered water molecules (O17). In the case of molecule A, the protein's C-terminus forms part of the substrate-binding pocket, sequestering the binding site from bulk solvent. In molecule B, the C-terminus is partially disordered. A similar partial disorder at the C-terminus was also observed in the structure of ligand-free HsaC, suggesting that crystal contacts may favor a more ordered conformation of the three C terminal residues (residue 298–300) in molecule A.

### Role of HsaC in cholesterol catabolism

**Figure 4:**
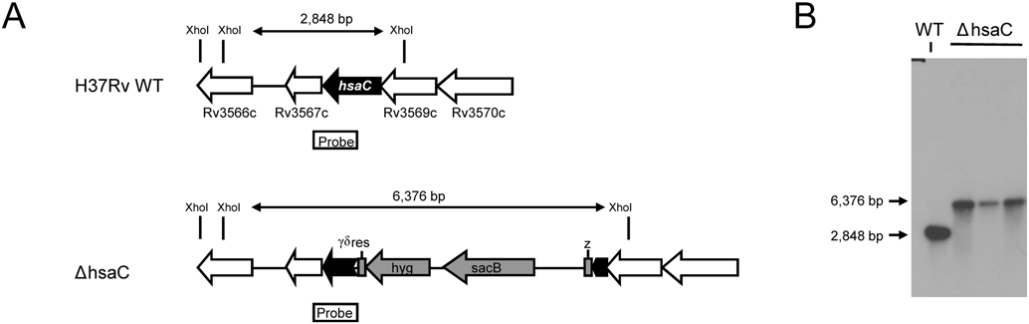
The design of a Δ*hsaC* mutant of *M. tuberculosis*. (A) Genetic organization of the *hsaC* locus in wild-type H37Rv and the Δ*hsaC* mutant. The size of the *Xho*I fragments as well as the location of the probe relevant for Southern analysis are indicated. γδ*res*, *res*-sites of the γδ-resolvase; *hyg*, hygromycin resistance gene. (B) Southern analysis of *Xho*I digested genomic DNA from wild-type H37Rv and three independent Δ*hsaC* mutant clones. Gene deletion was confirmed employing a [α-^32^P]dCTP-labeled probe hybridizing to the position indicated in A.

**Figure 5:**
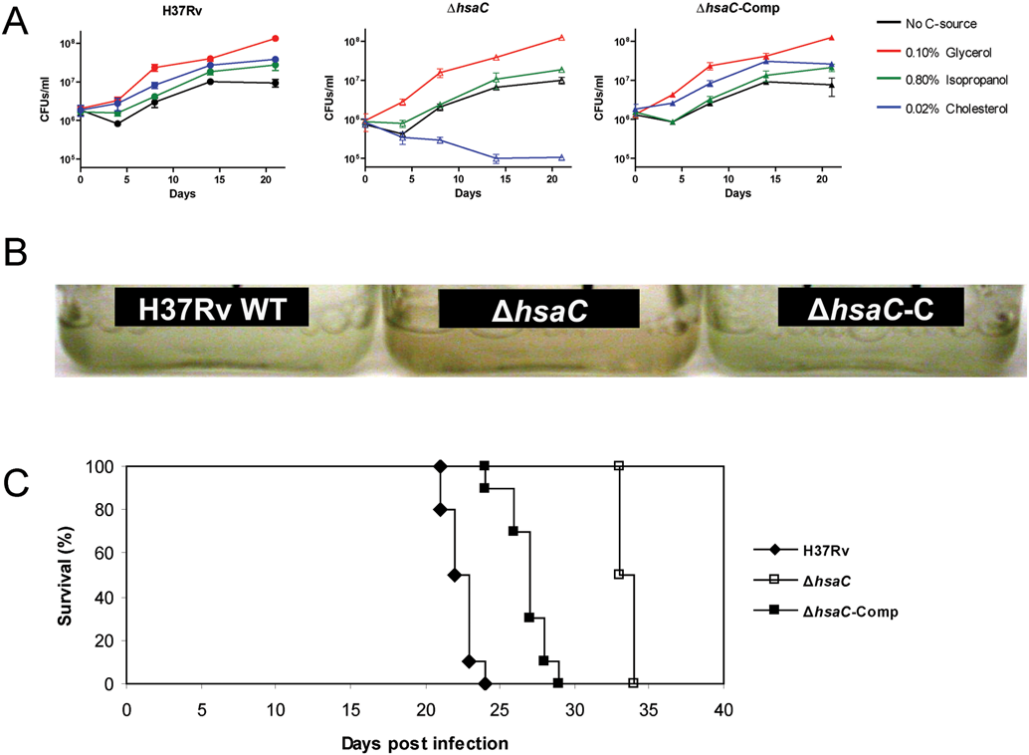
Growth of a Δ*hsaC* mutant of *M. tuberculosis* on cholesterol and in mice. (A) Growth of H37Rv strains in minimal media containing 0.1% (v/v) glycerol, 0.8% (v/v) isopropanol (solvent control), 0.02% (w/v) cholesterol with 0.8% (v/v) isopropanol, or no added carbon source. The plotted values represent the means of triplicates, with error bars indicating standard deviation. (B) Accumulation of a colored metabolite during cholesterol utilization by the Δ*hsaC* mutant. (C) Survival of SCID mice after intravenous infection with 10^5^ CFU of wild-type H37Rv, the Δ*hsaC* mutant or the complemented Δ*hsaC* mutant, respectively (*n* = 10 mice per group).

To assess the role of *HsaC* in cholesterol catabolism, we generated a precise null deletion mutant of *hsaC* in *M. tuberculosis* H37Rv by specialized transduction (Figure 4A and 4B). Growth on cholesterol and other organic substrates was tested using a minimal medium. This medium supported some background growth in the absence of added substrate. However, growth of wild-type H37Rv was measurably enhanced in the presence of cholesterol (Figure 5A), confirming that *M. tuberculosis* can utilize this steroid as a growth substrate. In contrast, the Δ*hsaC* mutant completely failed to grow on cholesterol while growth on glycerol was not impaired. Indeed, the Δ*hsaC* mutant displayed two notable phenotypes. First, the Δ*hsaC* mutant developed a pink color in the medium (Figure 5B), indicating the accumulation of catechols and their non-enzymatic oxidation to *o*-benzoquinones and condensation products, as observed in the Δ*hsaC* mutant of *R. jostii* RHA1 [4]. Second, the mutant lost viability in the presence of cholesterol, displaying a ten-fold decrease in CFU over 14-day growth experiment (Figure 5A).

### Role of cholesterol metabolism in pathogenesis

To evaluate the role of cholesterol metabolism in pathogenesis, we tested the Δ*hsaC* mutant in two animal models: immuno-compromised SCID mice and guinea pigs. Mice intravenously infected with 10^5^ CFU of the Δ*hsaC* mutant (median survival time 33.5 days±0.5 SD) survived substantially longer (p<0.0001, log-rank test) than those infected with wild-type H37Rv (median survival time 22.4 days±0.9 SD) or the complemented mutant Δ*hsaC attB*_L5_::pMV361::*hsaC* (median survival time 26.9 days±1.4 SD) (Figure 5C). These data corroborate the predicted importance of cholesterol catabolism for virulence of *M. tuberculosis* and emphasize the critical role of HsaC within this pathway. They further suggest that *M. tuberculosis* utilizes cholesterol early during infection, prior to the onset of adaptive immunity.

**Figure 6:**
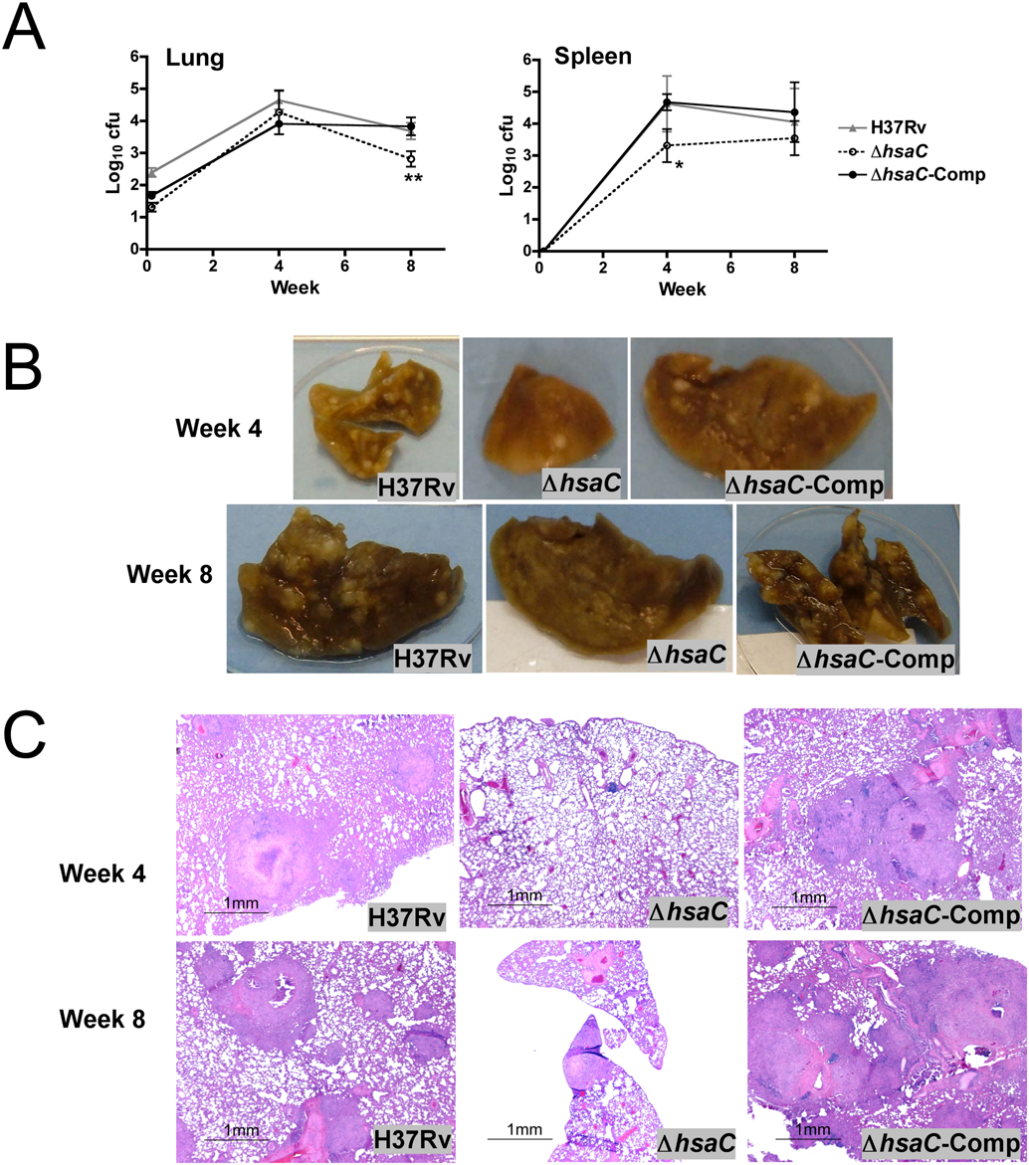
Growth of Δ*hsaC* mutant in the guinea pig model of tuberculosis. (A) Growth kinetics in the lung and spleen of guinea pigs aerosol-infected with H37Rv, Δ*hsaC* mutant, or the complemented Δ*hsaC* mutant (*n* = 15 guinea pigs per group). Asterisks indicate significant (*p<0.05) or highly significant (**p<0.01) differences found between guinea pigs infected with the H37Rv wild-type and Δ*hsaC* mutant strain. (B) Gross pathology of guinea pig lungs infected with wild-type, mutant, and complemented strains at week 4 and week 8. (C) Histopathological appearance of same lung specimens as those depicted in (B).

Guinea pigs infected via aerosol with ∼10^2^ CFU of the Δ*hsaC* mutant had similar bacillary loads in the lungs at 4 weeks post-infection as compared to both H37Rv and the complemented mutant strain. However at week 8, there were significantly fewer (p<0.01, two-way ANOVA) Δ*hsaC* organisms in the lung compared to either wild-type or complemented strains (Figure 6A). The spleens from the Δ*hsaC*-infected animals showed significantly (p<0.05) lower bacterial loads 4 weeks post-infection, suggesting an impaired dissemination to the organ. While implantation of the Δ*hsaC* mutant was slightly lower than the groups challenged with the wild-type and complemented strain (day 1), the differences were not statistically significant. In accordance with the CFU data, there were more grossly visible tubercles in lungs of animals infected with the wild-type or complemented strain compared to the mutant (Figure 6B). Microscopically, there were fewer lung granulomas (Table 3) at both week 4 (p<0.001) and week 8 (p<0.001) in mutant-infected guinea pigs (Figure 6C). Moreover, those in the Δ*hsaC*-infected guinea pig lungs were smaller and had less necrosis.

## Discussion

The phenotype of the Δ*hsaC* H37Rv mutant in cholesterol-containing medium, SCID mice and guinea pigs provides clear evidence that cholesterol metabolism contributes to the survival of *M. tuberculosis* in the host. The high specificity (*k*_cat_/*K*_m_) of HsaC for DHSA and the occupation of the enzyme's large, hydrophobic substrate-binding pocket with the bicyclo-alkanone moiety of the cholesterol metabolite are consistent with the enzyme's role in cholesterol metabolism, corroborating our previous demonstration that deletion of *hsaC* blocked growth on cholesterol in the related actinomycete, *R. jostii* RHA1 [4]. The first evidence for the role of cholesterol metabolism during pathogenesis was derived from genome-wide insertional mutagenesis studies [3] and the up-regulation of cholesterol catabolic genes during infection of macrophages [18]. Most recently, co-infection studies of mice using a mutant defective in cholesterol uptake indicated that cholesterol catabolism plays an important role in the chronic phases of infection [5].

The *in vitro* growth of *M. tuberculosis* on cholesterol and the loss of viability of the Δ*hsaC* mutant in the presence of cholesterol indicate that the attenuation of this mutant in the animal models is due to two factors: blockage of a catabolic pathway and the toxicity of catechols and/or quinones. The cytotoxicity of the latter compounds can arise from at least two mechanisms: (a) redox cycling between quinones and catechols to generate reactive oxygen species and (b) covalent modification of cellular components by the electrophilic *o*-benzoquinone [19]. This toxicity might be mitigated in the animal models by the fact that *M. tuberculosis* utilizes multiple growth substrates *in vivo*. Regardless of the precise mechanism of attenuation in the Δ*hsaC* mutant, the current data unambiguously establish that *M. tuberculosis* metabolizes cholesterol during infection. Moreover, the Δ*hsaC* mutant effectively provides a sensitive probe of the conditions under which cholesterol catabolism occurs, even when the latter is not essential.

The most striking result of the animal studies was the reduction in granulomas in guinea pigs infected with the Δ*hsaC* mutant. This is consistent with the conclusion of Pandey and Sassetti [5], and correlates with the recent finding of tubercule bacilli in close association with lipid droplets and crystalline cholesterol in a mouse model of caseating granulomas [20]. Indeed, histopathology studies have reported the progressive accumulation of cholesterol-rich lipid in alveolar macrophages leading to caseating granulomas in humans [21],[22]. Nevertheless, the current studies further indicate that cholesterol metabolism by *M. tuberculosis* contributes to bacillary multiplication during earlier stages of infection and to the dissemination of the pathogen in the host. The Δ*hsaC* mutant likely enabled detection of this effect due to the accumulation of a toxic metabolite. However, another difference between the studies is that the *mce4* permease mutant did not completely block growth on, nor metabolism of, cholesterol. Curiously, a different *mce4* mutant was much less attenuated [23]. Finally, the phenomenon of comparable bacillary counts accompanied by reduced lung pathology has been described for some sigma factors mutants and a *whiB3* mutant [24]. Nevertheless, it is unclear whether HsaC or an HsaC-dependent product is required for an inflammatory response in animal lungs while being dispensable for growth.

HsaC appears to be significantly more susceptible to oxidative inactivation during catalytic turnover than other characterized extradiol dioxygenases. For example, the partition ratios of HsaC for each of DHSA and DHDS are 50-fold less than that of BphC for its preferred substrate, DHB [13]. Some *meta*-cleavage pathways, such as the xylene catabolic pathway of *Pseudomonas putida* mt-2, have recruited a ferredoxin that reduces the catalytically essential iron of extradiol dioxygenases that is adventitiously oxidized during catalytic turnover, enabling the growth of the organism on a broader range of compounds [15]. BLAST searches indicate that the *M. tuberculosis* genome does not encode such a ferredoxin. This does not preclude the possibility that another reductase or electron-transfer protein plays this physiological role.

The susceptibility of HsaC to oxidative inactivation could reflect relatively low levels of O_2_ in *M. tuberculosis*-infected lungs. Tuberculous granulomas in lungs of guinea pigs, rabbits and non-human primates were found to be positive for the hypoxia marker pimonidazole hydrochloride (PIMO) [25] and the oxygen tension in small pulmonary lesions in infected rabbits were about 3% that of uninfected lungs and below the *K*_mO2_ of HsaC. Interestingly, hypoxic conditions have been shown to upregulate a number of genes in *M. tuberculosis*, including *fadD19*, an acyl CoA synthetase in the cholesterol metabolism pathway [26]. Although transposon mutagenesis studies have identified many cholesterol metabolic genes as essential for survival in activated macrophages [3], there is almost no correlation between the up-regulation of genes in response to low O_2_ and macrophage activation [18]. An intriguing possibility is that *M. tuberculosis* sequesters O_2_ for HsaC and other oxygenases of the pathway to improve the degradation of steroids in certain cellular environments. Indeed, trHbO, one of two truncated hemoglobins harbored by the pathogen and encoded by *glbO*, has been proposed to increase the availability of O_2_ for respiration [27]. Moreover, the heterologous expression of related hemoglobins increased the rate of the microbial degradation of aromatic compounds by dioxygenase-dependent pathways [28]. In a recent study, *glbO* was found to be most strongly up-regulated by hypoxia, and was also up-regulated late during infection of macrophages [29]. Nevertheless, the *K*_mO2_ of HsaC is almost two orders of magnitude greater than that of some extradiol dioxygenases isolated from hypoxic soil environments [30], suggesting that this enzyme, and by extension the cholesterol catabolic pathway of *M. tuberculosis*, has not evolved to function in extremely hypoxic environments.

The structure of the monodentate-bound HsaC:DHSA complex was unexpected, but potentially provides insights into the initial substrate-binding steps of extradiol dioxygenases. Observation of this species is reminiscent of the trapping of three catalytic intermediates in different protein molecules of a single crystal of HPCD [11], another extradiol dioxygenase. In that case, stabilization of different intermediates at different active sites was ascribed in part to crystal packing forces. In the current studies, the different packing forces affecting molecules A and B, reflected in the greater disorder of the C-terminal residues of molecule B, may contribute to stabilization of the different ligand binding mode. Irrespective of how the monodentate-bound catechol was stabilized, this species was proposed to occur by Groce *et al.*, who described the binding reaction of 4-nitrocatechol (4-NC) to HPCD as proceeding via three observable steps in addition to an unobserved initial rapid association step [12]. 4NC is a poor substrate for HPCD, binding to the active site as a dianion instead of as a monoanion observed for physiological substrates. Nevertheless, the initial binding of the catechol to the iron was proposed to be monodentate via the hydroxyl group attached to the ring carbon that is eventually subject to nucleophilic attack by the activated oxygen intermediate, consistent with the current structural data. Finally, the observation of the bicycloalkanone moiety of the DHSA in essentially identical positions (rmsd = 0.25 Å) in the bidentate and monodentate complexes suggests that this moiety is a determinant in the initial complex that is proposed to form reversibly between extradiol dioxygenases and their substrates.

Although 2′,6′-diCl DHB and 4-Cl DHDS efficiently inactivated HsaC during catalytic turnover, their respective modes of action likely differ, reflecting steric and electronic considerations, respectively. 2′,6′-DiCl DHB strongly inhibits BphC (*K*_ic_ = 7±1 nM) due to partial occlusion of the likely O_2_-binding site by one of the chloro substituents [7]. The O_2_-binding site, defined by Val148, Phe187 and Ala198 is conserved in HsaC (Val147, Phe192 and Ala203). 2′,6′-diCl DHB does not inhibit HsaC as effectively as BphC, likely due to the poorer fit of the non-hydroxylated phenyl ring into the active site (Figure S2). By contrast, 4-Cl DHDS likely inactivates HsaC due to the electron-withdrawing group on the catecholic ring. This basis of inactivation has been reported in a range of extradiol enzymes, including BphC [14] and human 3-hydroxy-anthranilate-3,4-dioxygenase [31], an enzyme essential to the biosynthesis of quinolinate from tryptophan. While the precise role of cholesterol metabolism by *Mtb* in human patients remains to be determined, particularly considering the limitations of the various animal models, the presented structural and kinetic data should facilitate the design of more potent inhibitors of HsaC.

## Materials and Methods

### Chemicals, strains, and growth

**Figure 7:**
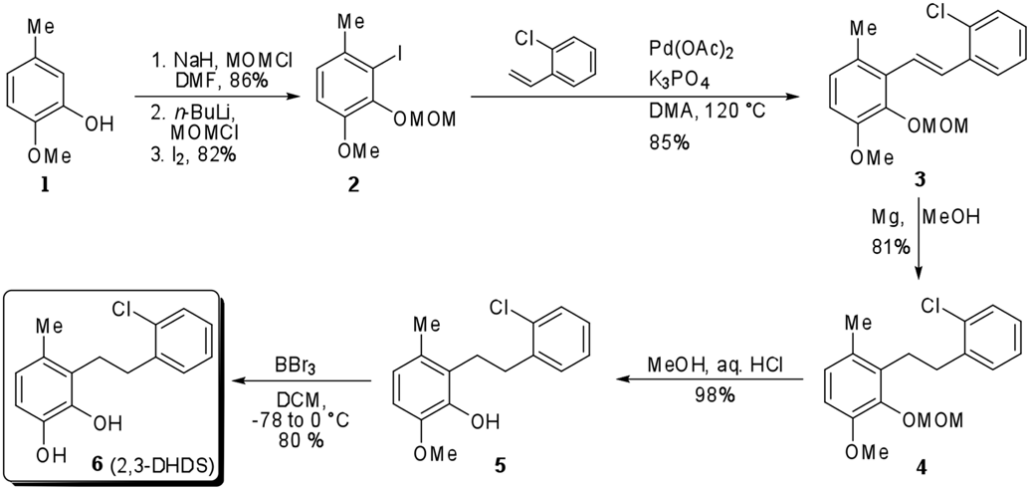
Preparation of 2,3-dihydroxy-6-methyl-7,8-dihydro-10-Cl-stilbene (DHDS) via directed *ortho* metalation.

DHB and 2′,6′-diCl DHB were synthesized according to established procedures [32]. DHDS (**6**; Figure 7) was prepared starting from commercially available 2-methoxy-5-methylphenol (**1**) which was converted into an intermediate MOM derivative allowing a directed *ortho* metalation (DoM) and iodination reaction sequence to form the corresponding aryl iodide **2**. Compound **2** was subjected to Heck coupling conditions, as precedented [33], to afford the stilbene **3** which was reduced (**4**) and deprotected (**5**) to give the requisite catechol **6**. The latter was purified by silica gel chromatography and its identity was confirmed by ^1^H and ^13^C NMR. ^1^H NMR of DHDS (400 MHz, CDCl_3_) δ/ppm: 7.37 (d, *J* = 7.1 Hz, 1H), 7.24–7.13 (m, 3H), 6.65 (d, *J* = 8.1 Hz, 1H), 6.60 (d, *J* = 8.1 Hz, 1H), 5.20 (s, 1H), 4.86 (*br* s, 1H), 2.93 (s, 4H), 2.23 (s, 1H). ^13^C NMR of DHDS (100 MHz, CDCl_3_) δ/ppm: 142.2, 141.0, 139.4, 133.8, 130.6, 129.5, 129.4, 127.5, 126.9, 126.4, 121.4, 112.6, 32.9, 27.1, 18.8. ^1^H NMR of 4-Cl DHDS (400 MHz, CDCl_3_) δ/ppm: 7.38–7.36 (m, 1 H), 7.20–7.16 (m, 3 H), 6.70 (s, 1 H), 5.52 (br s, 1 H), 5.35 (br s, 1 H), 2.96–2.94 (m, 4 H), 2.18 (s, 3 H). Full experimental details of the synthesis and characterization data of DHDS and related compounds will be presented in a future publication (J-X. Wang, L.D. Eltis, and V. Snieckus, unpublished results).

DHSA was generated by incubating a culture of the Δ*hsaC* mutant of *Rhodococcus jostii* RHA1 [4] with cholesterol. Briefly, several colonies were used to inoculate 100 ml W minimal salt medium [34] containing 20 mM pyruvate. At mid-log phase (OD_600_ of 1.0), 50 ml of preculture was used to inoculate 5 litres W media containing 20 mM pyruvate and 0.5 mM cholesterol. Cells are harvested at OD_600_ of 1.5 and pellet was resuspended in 0.5 litres W media containing 0.5 mM cholesterol in a 2-litre baffled flask. Production of metabolites in culture supernatant was monitored using HPLC. At highest DHSA production, the culture supernatant was collected by centrifugation, acidified using 0.5% orthophosphoric acid to ∼pH 6, and then extracted twice with 0.5 volumes of ethyl acetate. The ethyl acetate fractions were pooled, dried with anhydrous magnesium sulfate, and evaporated to dryness with a rotary evaporator. The residue was dissolved in a 44:56 mixture of methanol/water containing 0.5% phosphoric acid and purified using a Waters model 2695 HPLC (Milford, MA) equipped with a Prodigy 10-µm ODS-Prep column (4.6×250 mm; Phenomenex, Torrance, CA). Metabolites were eluted using the same methanol/water solvent at a flow rate of 1 ml/min. The eluate was monitored at 280 nm. Fractions containing DHSA (t_R_∼35 min) were pooled, added to 10 volumes of water, and extracted as described above. All other chemicals were of analytical grade or higher. HsaC from *M. tuberculosis* H37Rv was produced in *Escherichia coli*, as previously described [4]. *M. tuberculosis* H37Rv strains were grown in a minimal medium (KH_2_PO_4_ 1 g/l, Na_2_HPO_4_ 2.5 g/l, asparagine 0.5 g/l, ferric ammonium citrate 50 mg/l, MgSO_4_×7 H_2_O 0.5 g/l, CaCl_2_ 0.5 mg/l, ZnSO_4_ 0.1 mg/l, Tyloxapol 0.05%, v/v) containing 0.1% (v/v) glycerol or 0.02% (w/v) cholesterol. Cholesterol was added from a 25 mg/ml stock solution dissolved in isopropanol. Minimal medium containing 0.8% (v/v) isopropanol was used as a control. Growth was monitored by measuring colony forming units (CFU) by plating serial dilutions of cultures onto Middlebrook 7H10 agar supplemented with 10% (v/v) OADC enrichment (Becton Dickinson Microbiology Systems, Spark, MD) and 0.5% (v/v) glycerol.

### Generation of gene-deletion mutant

For generating an allelic exchange construct designed to replace the *hsaC* gene (Rv3568c) with a γδ*res*-*sacB*-*hyg*-γδ*res* cassette comprising the *sacB* and hygromycin resistance genes flanked by *res*-sites of the γδ-resolvase, upstream and downstream flanking DNA regions of *hsaC* were amplified by PCR employing the oligonucleotide pair Rv3568c-LL (5′-TTTTTTTTCCATAAATTGGTCCGCTGGTGGGCAAC TCGTT-3′) and Rv3568c-LR (5′-TTTTTTTTCCATTTCTTGGCCTTCGGCATTCGCGCATC-3′) introducing *Van*91I restriction sites (underlined) for amplification of the upstream flanking region Rv3568c-L, and the oligonucleotide pair Rv3568c-RL (5′-TTTTTTTTGCATAGATTGC AGCCGAGTGGTCAGCCCGTAT-3′) and Rv3568c-RR (5′-TTTTTTTTGCATCTTTTGCTAA CGGCGGTTCCAACGACA-3′) introducing *Bst*API restriction sites (underlined) for amplification of the downstream flanking region Rv3568c-R. Subsequently, Rv3568c-L and Rv3568c-R were digested with *Van*91I or *Bst*API, respectively, and ligated with *Van*91I-digested p0004S vector arms (T. Hsu and W.R. Jacobs Jr., unpublished results), resulting in the knock-out construct pRv3568cS which was then linearized with *Pac*I and cloned and packaged into the temperature-sensitive phage phAE159 (J. Kriakov and W.R. Jacobs Jr., unpublished results) as described [35], yielding the knock-out phage phRv3568cS. Allelic exchange in *M. tuberculosis* H37Rv using the phage phRv3568cS was achieved by specialized transduction as reported previously [35], resulting in deletion of nucleotides 220–375 of the *hsaC* gene (903 bp) and replacement by the γδ*res*-*sacB*-*hyg*-γδ*res* cassette (Figure 4A). The obtained mutants were verified by Southern analysis of *Xho*I-digested genomic DNA isolated from independent mutant clones as well as the wild-type using radiolabeled Rv3568c-R as probe (Figure 4B).

For complementation of the Δ*hsaC* mutant, the *hsaC* gene was amplified by PCR using the oligonucleotides 5′-TTTTTTCAGCTGCAATGAGCATCCGGTCGCTGGGC-3′ (5′ primer) and 5′-TTTTTTAAGCTTCTAGCCGCGAGCGCCTACGGTG-3′ (3′ primer) and cloned via the primer-introduced restriction sites (underlined) as a *Pvu*II-*Hin*dIII fragment downstream of the constitutive *hsp60* promoter into plasmid pMV361^KanR^, which allows single copy integration into the genome of *M. tuberculosis* [36] and complementation in *trans*, resulting in complemented mutant strain Δ*hsaC attB*_L5_::pMV361::*hsaC*.

### Animal infection studies

SCID/NCr (BALB/c background) mice (4- to 6-week-old females) were infected intravenously through the lateral tail vein with 10^5^ CFU of various *M. tuberculosis* H37Rv strains suspended in 200 µl PBS containing 0.05% Tween 80. Ten mice per group were infected and survival of mice was monitored.

Specific pathogen-free outbred Hartley strain guinea pigs (250–300 g; Charles River Breeding Laboratories, Inc. (Wilmington, MA)) were infected via the respiratory route in an aerosol chamber (University of Wisconsin Engineering Shops (Madison, WI)) with a nebulizer concentration of 2×10^7^ CFU/ml of the three strains of *M. tuberculosis* H37Rv [37] (n = 15 guinea pigs per group). Animals were euthanized on day 1, 4 weeks and 8 weeks post-infection. The right lower lung lobe and half of the spleen was homogenized in sterile saline and appropriate 10-fold dilutions were inoculated on M7H10 agar plates [38]. The lower left lung lobe and half of the spleen were taken for histopathology. Following 3 weeks of incubation at 37°C, the colonies were counted and the data were transformed in log_10_ values for statistical analysis. Mouse and guinea pig infection protocols were approved by the Animal Care and Use Committee at Albert Einstein College of Medicine and at Texas A&M University, respectively.

### Histopathology

The number of low power (20×) fields was counted for each specimen. Within each field, the number of granulomas was also tabulated permitting the calculation of the number of granulomas per low-power microscopic field. Because the size and extent of necrosis of each granuloma varies, a subjective determination on a scale of 1–4 of disease severity was also assessed so that both quantitative and qualitative measures could be used to describe the extent of tissue damage in a manner similar to a recently described method [39].

### Purification and kinetic characterization of HsaC

HsaC was purified anaerobically using a two-column protocol derived from that used to purify BphC [13]. Briefly, cells from 3 litres of culture were resuspended in 30 ml of 10 mM TRIS, pH 7.5 containing 1 mM MgCl_2_, 1 mM CaCl_2_ and 0.1 mg/ml Dnase I and disrupted using a French Press operated at 20,000 psi. The cell debris was removed by ultracentrifugation (120,000*g*×45 min). The clear supernatant fluid (∼40 ml) was decanted, referred to as the raw extract, and divided into two equal portions. Each portion was loaded onto a column packed with Source15 Phenyl resin (2×9 cm) and equilibrated with 10 mM TRIS, pH 7.5 containing 1 M ammonium sulphate. The column was operated at a flow rate of 5 ml/min. The enzyme activity was eluted with a linear gradient of 1 to 0 M ammonium sulphate over 8 column volumes. Fractions (10 ml) containing activity from the two runs were concentrated to 10 ml with a stirred cell concentrator equipped with a YM10 membrane (Amicon, Oakville, Ontario) and loaded onto a Mono Q anion exchange column (1×8 cm) equilibrated with 10 mM TRIS, pH 7.5 containing 5% *t*-butanol, 2 mM dithiothreitol (DTT) and 0.25 mM ferrous ammonium sulphate. The column was operated at a flow rate of 2 ml/min. The enzyme activity was eluted with a linear gradient of 0.2 to 0.4 M NaCl over 20 column volumes. Fractions exhibiting activity were combined, exchanged into the column equilibration buffer, concentrated to 20–25 mg/ml protein, and flash frozen as beads in liquid N_2_. Purified HsaC was stored at −80°C for several months without significant loss of activity. Aliquots of HsaC were thawed immediately before use and exchanged into 20 mM HEPES, 80 mM NaCl (I = 0.1), pH 7.0 containing 5% *t*-butanol using a desalting column. Samples of HsaC were further diluted for enzyme kinetics using the same buffer containing 0.1 mg/ml BSA and 2 mM DTT, except in the inactivation experiments. For the latter, enzyme was diluted in the same buffer without DTT and were used within two hours. HsaC activity was verified at the beginning and end of each set of experiments. Protein and iron concentrations were evaluated colorimetrically using the Bradford method [40] and Ferene S [41], respectively.

Enzyme activity was routinely measured by following the consumption of O_2_ using a Clark-type polarographic electrode as described previously [13] unless otherwise stated. Experiments were performed in a total volume of 1.3 ml 20 mM HEPES, 80 mM NaCl, pH 7.0, 25.0±0.1°C equilibrated with 5% O_2_ in N_2_ (103±3 µM dissolved O_2_). Reaction buffers containing different concentrations of dissolved O_2_ were prepared by bubbling them with mixtures of O_2_ and N_2_ gases and transferring them to the reaction chamber as described previously [13]. The amount of active HsaC was defined by the iron content of the sample. Steady-state kinetic parameters were calculated using LEONORA [42].

Cleavage of 2′6′-diCl DHB and was measured by following the rate of appearance of the ring-cleaved product using a Cary 5000 spectrophotometer equipped with a thermojacketed cuvette holder (Varian, Walnut Creek, CA). Initial velocities were determined from a least-squares analysis of the linear portion of the progress curves. Partition ratios expressing the number of substrate molecules consumed per enzyme molecule inactivated were determined spectrophotometrically for DHB, 2′,6′-diCl DHB, DHDS, and DHSA by following the appearance of the ring-cleaved products at 434 nm (ε = 23.4 mM^−1^ cm^−1^), 391 nm (ε = 36.5 mM^−1^ cm^−1^), 396 nm (ε = 6.3 mM^−1^ cm^−1^), and 392 nm (ε = 7.6 mM^−1^ cm^−1^), respectively. The partition ratio for 4-Cl DHDS was determined by oxygraph electrode due to a very low extinction coefficient. Partition ratios were determined under saturating substrate conditions ([S]≫K_m_).

### Crystallization and preparation of complex

Crystals of substrate-free HsaC were grown anaerobically at room temperature using the hanging drop method (protein 15 mg/ml, crystallization solution: 12–15% PEG 3350, 0.2 M ammonium tartrate, 25% ethylene glycol). Single crystals appeared in 2–5 days and grew to their full size (200 µm×200 µm) in two weeks. The crystals were frozen anaerobically in liquid N_2_ prior to diffraction experiments. The complex with DHSA was formed by adding 0.2–0.5 µl of crude extract containing DHSA dissolved in *t*-butanol directly to the drop containing the crystal and incubating for up to 2 hr (anaerobically) at room temperature. The HsaC:DHSA crystals were flash frozen in liquid N_2_.

### Diffraction experiments and structure analysis

X-ray data collections were performed under cryogenic conditions using an in-house rotating anode X-ray generator (CuKα radiation, λ = 1.542 Å) and at the Advanced Light Source (ALS, Beamline 8.2.2). Data were processed using HKL2000 [43]. Molecular replacement was performed using PHASER [44] and the structure of substrate-free BphC (PDB accession code 1HAN) with Fe and waters deleted as a search model. The highest scoring solution placed a dimer in the asymmetric unit, which was used as a starting model for re-building and structure refinement, which was performed using CNS [45] (simulated annealing) and REFMAC [46] in alternation with manual rebuilding using COOT [47].

For the HsaC:DHSA complexes, difference Fourier electron density maps revealed additional density within the active site consistent with a bound DHSA. The diffraction data and properties of the refined model are characterized in Table 2. A model for the substrate was established using the PRODRG server. Electron density maps were calculated with the CCP4 suite (FFT function). Structural figures and graphical rendering were made by using PYMOL [48]. The final model of HsaC:DHSA contains a dimer of HsaC covering 299/298 residues of each chain, two DHSA molecules, and 485 water molecules. The final model of substrate-free HsaC contains a dimer of HsaC covering 295 residues (2–296) of each chain, one tartrate and 712 water molecules. The coordinates for HsaC:DHSA and HsaC alone were deposited in the Protein Data Bank (www.pdb.org) with accession codes 2ZI8 and 2ZYQ, respectively.

## Supporting Information

Figure S1 Electron density corresponding to the two binding modes of DHSA in HsaC. (A) DHSA bidentate bound. (B) DHSA monodentate bound. The upper and middle panels show simple electron density omit-maps (black) calculated using phases derived from the HsaC model without ligands. For clarity purposes the ligand (in ball-and-stick) was included in the upper panel figure. The omit-maps are contoured at 0.7 σ; roughly half of the mean electron density level of the surrounding protein structure. The bottom panel shows a F_o_−F_c_ electron density from restrained refinement (black, contour level = 3 σ), performed without ligands in the model.
(2.01 MB PDF)

Figure S2 Structural superposition of HsaC:DHSA and BphC:DHB. The respective catecholic rings of DHSA (C atoms colored yellow) and DHB (C atoms colored dark grey) bind in similar positions, while the remaining part of the substrates assume different orientations. Residues of HsaC (C atoms colored green) are labeled. Residues stabilizing the bicycloalkanone moiety of DHSA (Leu174, Leu190, Leu205, Val214 and Phe294) are conserved in extradiol dioxygenases known or thought to preferentially cleave DHSA.
(0.12 MB PDF)

Table S1 Fe-Ligand distances for the HsaC:DHSA monodentate^B^ and bidentate^A^ complexes as well as in the ligand-free form.
(0.03 MB DOC)

Table S2 Ligand-Fe-Ligand angles for the HsaC:DHSA monodentate and bidentate complexes as well as in the ligand-free form.
(0.04 MB DOC)
