# Supplementary figures and images for: Studies of a Ring-Cleaving Dioxygenase Illuminate the Role of Cholesterol Metabolism in the Pathogenesis of Mycobacterium tuberculosis
Source: PLoS Pathog. 2009 Mar 20;5(3):e1000344. doi: 10.1371/journal.ppat.1000344 (PMC2652662; doi:10.1371/journal.ppat.1000344)

**Figure S1**

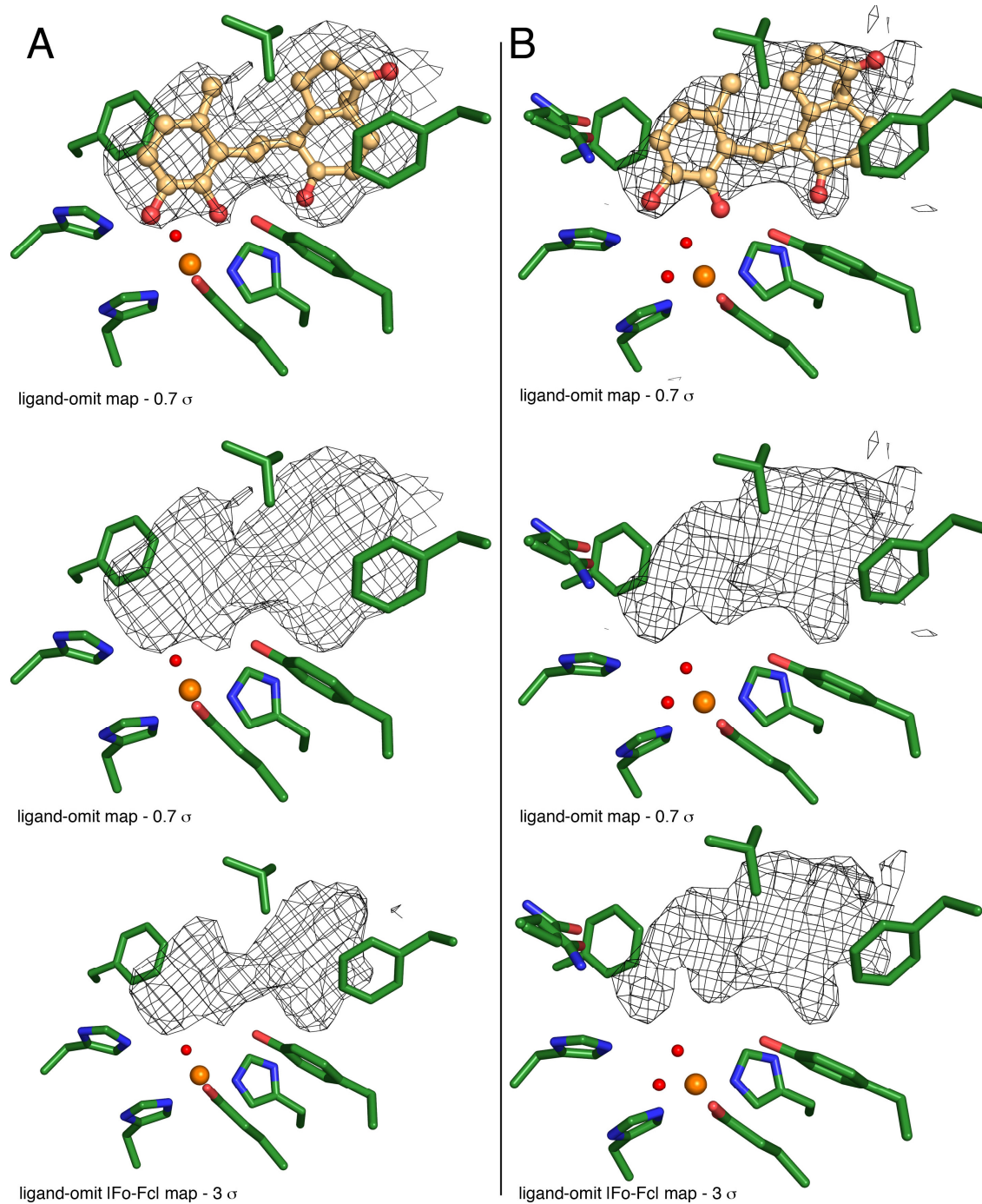

Supplement: Figure S1 — Electron density corresponding to the two binding modes of DHSA in HsaC. (A) DHSA bidentate bound. (B) DHSA monodentate bound. The upper and middle panels show simple electron density omit-maps (black) calculated using phases derived from the HsaC model without ligands. For clarity purposes the ligand (in ball-and-stick) was included in the upper panel figure. The omit-maps are contoured at 0.7 σ; roughly half of the mean electron density level of the surrounding protein structure. The bottom panel shows a Fo−Fc electron density from restrained refinement (black, contour level = 3 σ), performed without ligands in the model. (2.01 MB PDF) [file ppat.1000344.s001.pdf]

**Figure S2**

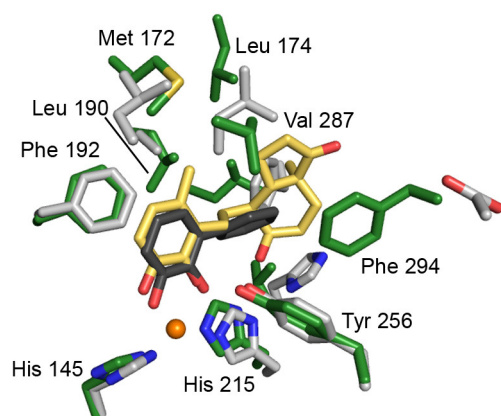

Supplement: Figure S2 — Structural superposition of HsaC:DHSA and BphC:DHB. The respective catecholic rings of DHSA (C atoms colored yellow) and DHB (C atoms colored dark grey) bind in similar positions, while the remaining part of the substrates assume different orientations. Residues of HsaC (C atoms colored green) are labeled. Residues stabilizing the bicycloalkanone moiety of DHSA (Leu174, Leu190, Leu205, Val214 and Phe294) are conserved in extradiol dioxygenases known or thought to preferentially cleave DHSA. (0.12 MB PDF) [file ppat.1000344.s002.pdf]
