# Supplementary material for: Studies of a Ring-Cleaving Dioxygenase Illuminate the Role of Cholesterol Metabolism in the Pathogenesis of Mycobacterium tuberculosis
Source: PLoS Pathog. 2009 Mar 20;5(3):e1000344. doi: 10.1371/journal.ppat.1000344 (PMC2652662; doi:10.1371/journal.ppat.1000344)
Supplement: Table S1 — Fe-Ligand distances for the HsaC:DHSA monodentateB and bidentateA complexes as well as in the ligand-free form. (0.03 MB DOC) [file ppat.1000344.s003.doc]

**Table S1**: Fe-Ligand distances for the HsaC:DHSA monodentate^B^ and bidentate^A^ complexes as well as in the ligand-free form.

| Metal-ligand distances (Å) | | | |
| --- | --- | --- | --- |
| Defining atom | HsaC | HsaC:DHSA^A^ | HsaC:DHSA^B^ |
| Fe- Nε2, H145 | 2.1 | 2.1 | 2.2 |
| Fe- Nε2, H215 | 2.2 | 2.1 | 2.1 |
| Fe- Oε2, E266 | 2.0 | 2.0 | 1.9 |
| Fe-Wat1 | 2.1 |  |  |
| Fe-Wat2 | 2.1 |  |  |
| Fe-O4 |  | 2.3 | 2.8 |
| Fe-O3 |  | 2.6 | (3.7) |
| Fe-Wat267 |  | 1.7 |  |
| Fe-Wat485 |  |  | 1.6 |
| Fe-Wat373 |  |  | 2.3 |
