# Supplementary material for: Studies of a Ring-Cleaving Dioxygenase Illuminate the Role of Cholesterol Metabolism in the Pathogenesis of Mycobacterium tuberculosis
Source: PLoS Pathog. 2009 Mar 20;5(3):e1000344. doi: 10.1371/journal.ppat.1000344 (PMC2652662; doi:10.1371/journal.ppat.1000344)
Supplement: Table S2 — Ligand-Fe-Ligand angles for the HsaC:DHSA monodentate and bidentate complexes as well as in the ligand-free form. (0.04 MB DOC) [file ppat.1000344.s004.doc]

**Table S2**: Ligand-Fe-Ligand angles for the HsaC:DHSA monodentate and bidentate complexes as well as in the ligand-free form.

| Ligand-metal-ligand angles (degrees) | | |
| --- | --- | --- |
| Substrate-free | Molecule A (bidentate) | Molecule B (monodentate) |
| 145-Fe-215 101 | 145-Fe-215 101 | 145-Fe-215 101 |
| 145-Fe-266 112 | 145-Fe-266 106 | 145-Fe-266 110 |
| 145-Fe-Wat1 97 | 145-Fe-Wat267 84 | 145-Fe-Wat373 89 |
| 145-Fe-Wat2 98 | 145-Fe-O3 99 | 145-Fe-Wat485 88 |
| 215-Fe-Wat1 87 | 145-Fe-O4 144 | 145-Fe-O4 136 |
| 215-Fe-Wat2 160 | 215-Fe-266 100 | 215-Fe-266 101 |
| 215-Fe-266 89 | 215-Fe-Wat267 91 | 215-Fe-Wat373 168 |
| 266-Fe-Wat1 150 | 215-Fe-O3 137 | 215-Fe-Wat485 94 |
| 266-Fe-Wat2 87 | 215-Fe-O4 73 | 215-Fe-O4 81 |
| Wat1-Fe-Wat2 84 | 266-Fe-O3 110 | 266-Fe-Wat373 72 |
|  | 266-Fe-O4 109 | 266-Fe-Wat485 151 |
|  | 266-Fe-Wat267 162 | 266-Fe-O4 111 |
|  | Wat267-Fe-O3 53 | Wat373-Fe-O4 92 |
|  | Wat267-Fe-O4 61 | Wat373-Fe-Wat485 87 |
|  | O3-Fe-O4 68 | Wat485-Fe-O4 48 |
